# Genomic diversity of uropathogenic *Escherichia coli* in clinical isolates from six latin american countries, 2018-2023

**DOI:** 10.17843/rpmesp.2025.422.14299

**Published:** 2025-06-19

**Authors:** Francesca Caballero, Anne Martinez-Ventura, Diego Cuicapuza, Alex Fajardo-Loyola, Rosmery Gutierrez-Ajalcriña, Javier Soto-Pastrana, Percy Asmat-Marrufo, Evelyn Barco-Yaipen de Vera, Henry Meza-Fernandez, Mario Chambi-Quispe, Jimena Pino-Dueñas, Nicomedes Laura-Rivas, Alexander Briones-Alejo, Pilar Diaz-Rengifo, Carlos Peralta-Siesquen, Guillermo Salvatierra, Pablo Tsukayama, Pool Marcos-Carbajal

**Affiliations:** 1 Cayetano Heredia Peruvian University, Microbial Genomics Laboratory, Lima, Peru. Cayetano Heredia Peruvian University Cayetano Heredia Peruvian University Microbial Genomics Laboratory Lima Peru; 2 Emerge (Emerging Diseases and Climate Change Research Unit), School of Public Health and Administration, Cayetano Heredia Peruvian University, Lima, Peru. Cayetano Heredia Peruvian University Emerge (Emerging Diseases and Climate Change Research Unit) School of Public Health and Administration Cayetano Heredia Peruvian University Lima Peru; 3 School of Medicine, Cayetano Heredia Peruvian University, Lima, Peru. Cayetano Heredia Peruvian University School of Medicine Cayetano Heredia Peruvian University Lima Peru; 4 National Institute of Health, National Center for Public Health, Laboratory of Biotechnology and Molecular Biology, Lima, Peru. National Institute of Health, National Center for Public Health Laboratory of Biotechnology and Molecular Biology, Lima Peru; 5 Huaycán Hospital, Epidemiology Department, Lima, Peru. Huaycán Hospital Epidemiology Department Lima Peru; 6 Mother and Child San Bartolomé National Teaching Hospital, Department of Clinical Pathology, Microbiology Unit, Lima, Peru. Mother and Child San Bartolomé National Teaching Hospital Department of Clinical Pathology Microbiology Unit Lima Peru; 7 Laboratorio de Referencia Regional Salud Pública, Servicio de microbiología, Trujillo, La Libertad, Perú. Laboratorio de Referencia Regional Salud Pública Servicio de microbiología Trujillo La Libertad Peru; 8 JAMO Hospital, Microbiology Department, Tumbes, Peru. JAMO Hospital Microbiology Department Tumbes Peru; 9 Alberto Sabogal Sologuren Hospital, Department of Clinical Pathology, Microbiology Service, Bellavista, Callao, Peru. ">Alberto Sabogal Sologuren Hospital Department of Clinical Pathology Microbiology Service Bellavista Callao Peru; 10 Carlos Monge Medrano Hospital, Clinical Pathology, Puno, Peru. Carlos Monge Medrano Hospital Clinical Pathology Puno Peru; 11 Cusco Regional Hospital, Clinical Pathology, Cusco, Peru. Cusco Regional Hospital Clinical Pathology Cusco Peru; 12 Huancavelica Regional Public Health Reference Laboratory, Microbiology Service, Huancavelica, Peru. Huancavelica Regional Public Health Reference Laboratory Microbiology Service Huancavelica Peru; 13 Loreto Regional Hospital, Microbiology Department, Iquitos, Peru. Loreto Regional Hospital Microbiology Department Iquitos Peru; 14 San Martín Regional Public Health Referral Laboratory, Microbiology Service, Tarapoto, Peru. San Martín Regional Public Health Referral Laboratory Microbiology Service Tarapoto Peru; 15 Jorge Chávez IPRESS, Microbiology Department, Madre de Dios, Peru. Jorge Chávez IPRESS Microbiology Department Madre de Dios Peru; 16 Alexander von Humboldt Institute of Tropical Medicine, Cayetano Heredia Peruvian University, Lima, Peru. Cayetano Heredia Peruvian University Alexander von Humboldt Institute of Tropical Medicine Cayetano Heredia Peruvian University Lima Peru; 17 Union Peruvian University, Professional School of Medicine, Molecular Biology Research Laboratory, Lima, Peru. Union Peruvian University Union Peruvian University Professional School of Medicine Molecular Biology Research Laboratory Lima Peru

**Keywords:** Escherichia coli, Uropathogen, UPEC, Bacterial Resistance, Molecular Epidemiology

## Abstract

**Objective.:**

To genetically characterize clinical isolates of uropathogenic *Escherichia coli* (UPEC) from hospitals in Peru and contextualize them against 127 additional UPEC genomes reported in six Latin American countries between 2018 and 2023.

**Materials and methods.:**

The genomes of 16 Peruvian UPEC isolates were sequenced, assembled and supplemented with 127 genomes available in the NCBI public database. Serotypes, sequence types (STs), antimicrobial resistance (AMR) genes, and resistance-associated mutations were identified. A phylogenetic analysis was also conducted in order to determine evolutionary relations and distribution in phylogroups.

**Results.:**

The ST131 clone was the most prevalent (42.7%), followed by ST1193 (13.3%). Phylogroup B2 was widely predominant (83.2%), with serotype O25:H4 standing out. The resistance genes *blaTEM-1*, *blaCTX-M-15*, and *blaCTX-M-27* were identified with high frequency, as well as mutations in *gyrA* and *parC* associated with fluoroquinolone resistance, especially in the ST131 clone.

**Conclusion.:**

Our findings show high circulation of high-risk UPEC clones, such as ST131 and ST1193, in Latin America, along with a notable burden of genes and mutations linked to multidrug resistance, highlighting the need to strengthen regional genomic surveillance.

## INTRODUCTION

*Escherichia coli* is a Gram-negative bacillus of the *Enterobacteriaceae* family that is part of the normal intestinal microbiota in most humans. However, there are opportunistic variants, known as uropathogenic *E. coli* (UPEC), capable of colonizing the urinary tract and causing infections ^1^^,^^2^. These UPEC strains are characterized by the presence of at least three specific virulence factors: *chuA* and *fyuA*, involved in iron acquisition; *vat*, which encodes a vacuolization-inducing autotransporting toxin; and *yfcV*, associated with fimbriae that facilitate adhesion to urothelial cells ^3^.

UPEC are the leading cause of urinary tract infections (UTIs) worldwide, affecting approximately 400 million people per year and causing nearly 230,000 deaths annually, according to the 2019 Global Burden of Disease study ^4^. These infections are the second most common type in adults ^5^ and are classified as complicated (cUTI) when there are comorbidities (pregnancy, immunocompromised status) or urinary tract abnormalities (obstruction, hydronephrosis, kidney stones), and uncomplicated (uUTI) in the absence of these conditions ^6^. Antibiotics such as ampicillin, sulfamethoxazole, and ciprofloxacin are used to treat uUTIs, while nitrofurans, cephalosporins, and carbapenems are recommended for cUTIs ^5^.

The inappropriate use and poor regulation of broad-spectrum antibiotics have contributed significantly to the increase in antimicrobial resistance (AMR), particularly in low- and middle-income countries ^7^. Regarding UPEC, the most important AMR mechanisms include the production of extended-spectrum beta-lactamases (ESBLs), enzymes capable of hydrolyzing penicillin, beta-lactams, and cephalosporins ^8^. Among these enzymes, *blaCTX-M-1* stands out, which is capable of hydrolyzing third-generation cephalosporins and one of the most common beta-lactamases in ESBL-producing *E. coli*^9^. In addition, there are non-enzymatic resistance mechanisms, such as point mutations in target genes*: parC/E* and *gyrA/B* are associated with resistance to fluoroquinolones ^10^, *glpT* and *uhpT* with fosfomycin ^11^, and *pmrA/B/D* with colistin ^12^. Other non-enzymatic mechanisms include efflux pumps, such as those encoded by the *emrD*, *acrF*, and *mdtM* genes, which are related to multidrug resistance (MDR) ^13^. Between 2000 and 2019, in Europe, Asia, and America, high phenotypic resistance to quinolones (49.4%), beta-lactams (36.9%), aminoglycosides (28.7%), and fosfomycin (8.4%) was reported ^14^.

Advances in genomics in recent decades now allow for the precise identification of sequence types (STs) and high-risk clones. Among these, ST131 stands out as the MDR clone with the highest risk worldwide ^15^. In Australia, in 2013, ST131 (27%) and serotype O25b (85%) were found to be the most frequent in urinary isolates from a sample of women of reproductive age ^16^. In 2022, in the United States, 23% of *E. coli* isolates belonged to the emerging pandemic clone ST1193, characterized by its resistance to fluoroquinolones, and its prevalence was 51% in China ^17^.

The lack of information on the genomic diversity and resistance patterns of UPEC in Latin America hinders its monitoring and treatment. Most studies are limited to identifying lineage markers and resistance genes using conventional PCR. Although whole genome sequencing is a key tool for high-resolution, large-scale characterization of isolates, to date few studies in the region have reported using this technique for UPEC. Therefore, in order to better understand the genomic diversity of this pathogen in Latin America, this study aimed to characterize 16 UPEC isolates from hospitals in Peru and analyze them in the context of 127 UPEC genomes reported in six countries in the region between 2018 and 2023.

**KEY MESSAGES**

Motivation for the study. To contribute to the genomic surveillance of UPEC in clinical samples from Latin America, in response to the growing public health problem represented by UTIs and their resistance to antimicrobials.

Main findings. Our study revealed a high frequency of high-risk clones, such as ST131 and ST1193. Critical mutations were identified in genes associated with resistance to multiple antibiotics, including fluoroquinolones, beta-lactams, and fosfomycin.

Implications. Our results highlight the urgent need to strengthen UPEC surveillance in Latin America. Tracking resistant strains and implementing measures to limit their spread is crucial and has a significant impact on the effectiveness of available treatments.

## MATERIALS AND METHODS

### Study design

**Figure 1:**
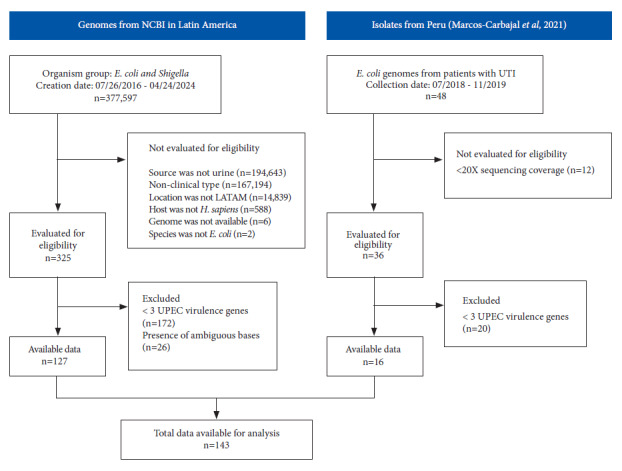
Flowchart showing the search for uropathogenic E. coli (UPEC) genomic sequences in the NCBI Isolates Browser database in Latin America and genomes from isolates from patients with UTI in Peru. A total of 127 genomes from Latin America were included from NCBI and supplemented with 16 collected from hospitals in Peru, with a total of 143 UPEC genomes available for analysis.

We conducted a descriptive study. The study population consisted of 143 UPEC isolates from Latin America. Of these, 127 correspond to genomes from the NCBI public database and 16 from outpatients with a clinical diagnosis compatible with urinary tract infection (UTI) in hospitals in Peru (Figure 1).

NCBI: National Center for Biotechnology Information, LATAM: Latin America, UPEC: uropathogenic *E. coli*, UTI: urinary tract infection.

### Genomic sequencing of UPEC isolates in Peru

**Figure 2:**
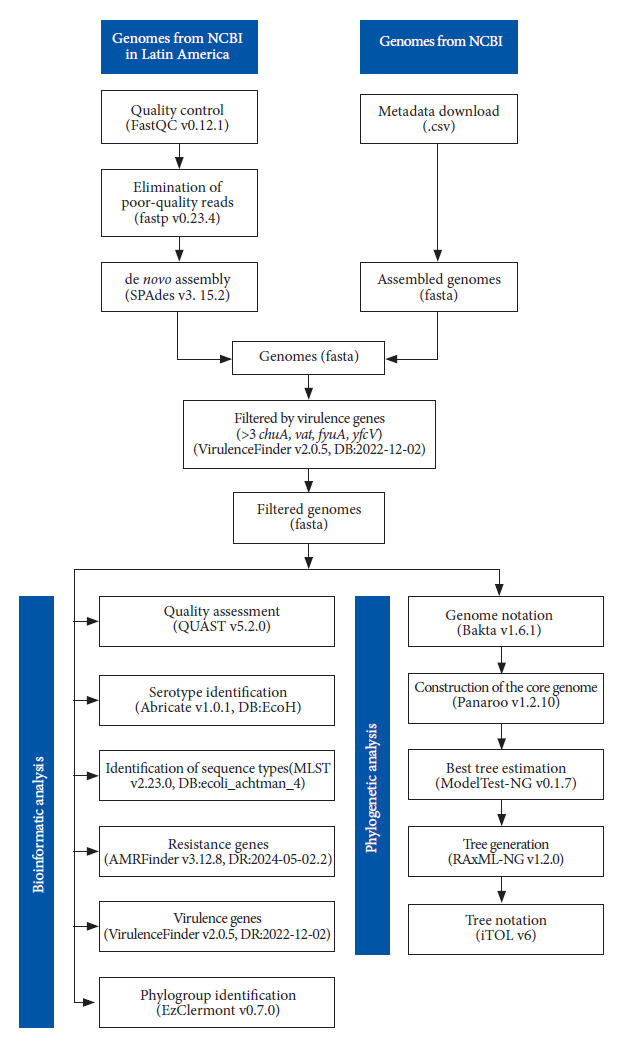
Flowchart of the bioinformatic and phylogenetic analysis. Reads from patients isolated from hospitals in Peru were assembled and processed together with genomes from NCBI using typing and phylogeny tools.

*E. coli* strains previously characterized phenotypically were collected from patients with UTI in eight Peruvian hospitals during 2018-2019 ^18^. Total DNA was extracted from 5 ml of liquid culture in TSB medium using the Thermo GeneJet Genomic DNA Purification Kit (Thermo Scientific) in a final volume of 100 µl. DNA quantification was performed with the Qubit 4 fluorometer and the dsDNA HS kit (Thermo Scientific). From the 200 original isolates, 48 were selected for sequencing using stratified sampling, prioritizing diversity in antimicrobial resistance profiles and geographical representativeness of the collection sites (Figure 1). Genomic libraries were prepared with the NexteraXT kit (Illumina) and sequenced on an Illumina MiSeq instrument at UPCH, using MiSeq v2 500-cycle kits. The raw sequences obtained from sequencing (Fastq format) were subjected to quality control using FastQC v0.12.1 (https://github.com/s-andrews/FastQC). Subsequently, the fastp v0.23.4 program (https://github.com/OpenGene/fastp) was used to remove adapters and low-quality sequences (Q<30 and length <50 bp), generating paired R1 and R2 files. Finally, the processed reads were assembled *de novo* using default parameters with SPAdes v3.15.2 (https://github.com/ablab/spades). The quality of the assembled genomes was evaluated using the QUAST v5.2.0 tool (https://github.com/ablab/quast) (Figure 2). The fastq and fasta files were deposited in the following BioProject: PRJNA1153025.

### Public UPEC genomes in Latin America

Genomes assembled in Fasta format were downloaded from the NCBI Isolates Browser (https://www.ncbi.nlm.nih.gov/pathogens/isolates/), along with associated metadata, which included relevant information such as the isolate identifier code, assembly code, year of collection, and country of origin. The selection criteria for the search included the taxonomic group (taxgroup_name) “*E. coli* and *Shigella*”; the isolate type (epi_type) “clinical”; the isolate source (isolation_source) ‘urine’; the host (host) “*Homo sapiens*”; and the geographical location (geo_loc_name) limited to “Paraguay,” Argentina, Colombia, Brazil, Chile, Peru, Mexico, Bolivia, Costa Rica, Cuba, Ecuador, El Salvador, Guatemala, Haiti, Honduras, Nicaragua, Panama, Dominican Republic, Uruguay, and Venezuela, in the period between 2016 and 2024. A total of 325 public genomes met these criteria (Figure 1).

### Refinement of UPEC genome selection

To confirm that the *E. coli* isolates corresponded to the UPEC type, genomes possessing three or more of the virulence factors proposed by Spurbeck *et al*. ^3^*chuA*, *vat*, *fyuA*, and *yfcV* were included, indicating a genetic profile consistent with UPEC. In addition, quality filters were applied, requiring sequences to be between 4.5 and 5.5 million base pairs (Mb) in length, and genomes with ambiguous bases were excluded. After applying these filters, the final number of considered public genomes was 127, to which we added 16 UPEC genomes generated in this study, resulting in a total of 143 genomes analyzed (Figure 1).

### Classification, gene identification, and phylogenetic analysis

To identify the serotype of the isolates, we used the ABRicate v1.0.1 tool (https://github.com/tseemann/abricate) with the EcOH database. Sequence types (STs) were determined according to the ecoli_achtman_4 scheme, derived from seven marker genes, using the MLST v2.23.0 program (https://github.com/tseemann/mlst). Antimicrobial resistance genes and mutations were identified using AMRFinderPlus v3.12.8 (https://github.com/ncbi/amr, database 2024-05-02.2). Virulence genes were identified using VirulenceFinder v2.0.5 (database 2022-12-02) available on the Center for Genomic Epidemiology (CGE) server (https://cge.food.dtu.dk/services/VirulenceFinder/). In addition, the phylogroup was identified according to the Clermont scheme using EzClermont v0.7.0 (https://github.com/nickp60/EzClermont). All analyses used a cutoff threshold of 90% for coverage and identity. The assembled genomes were annotated using Bakta v1.6.1 (https://github.com/oschwengers/bakta) and core genome alignment was performed with Panaroo v1.2.10 (https://github.com/gtonkinhill/panaroo). Subsequently, the best model for constructing the phylogenetic tree was determined using ModelTest-NG v0.1.7 (https://github.com/ddarriba/modeltest). Finally, the tree was constructed using the GTR+I+G4 model and performing 100 bootstrap replicates with RAxML-NG v1.2.0 (https://github.com/amkozlov/raxml-ng). An *Escherichia fergusonii* genome (CP083638.1) was used to root the tree. The tree was annotated and visualized using iTOL v6 (https://itol.embl.de/) (Figure 2).

### Ethical considerations

This study was evaluated and approved by the Ethics Committee of the Universidad Peruana Unión (N2019-CEUPeU-0001) and by the Institutional Committee on Research Ethics (CIEI) of the Universidad Peruana Cayetano Heredia (SIDISI No. 214524 and No. 214927). Only bacterial isolates were used, without including or analyzing any personal or clinical information about the patients.

## RESULTS

**Figure 3:**
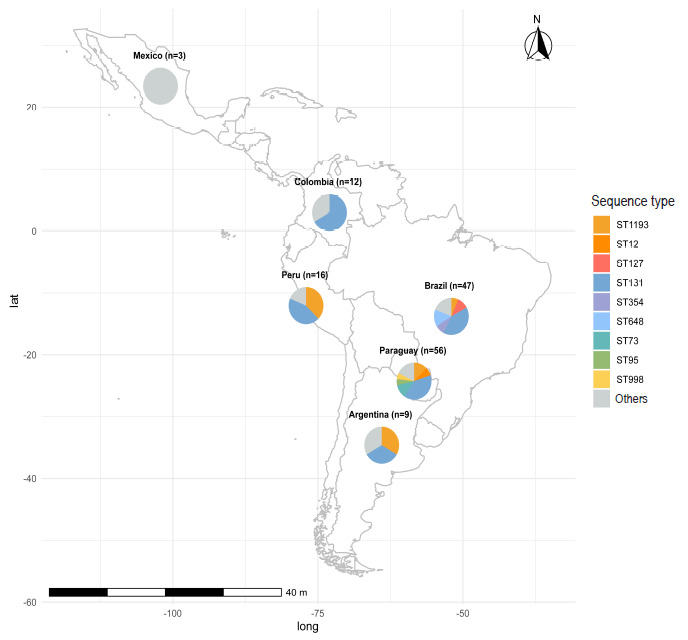
Geographic distribution of uropathogenic *E. coli* (UPEC) clones in Latin America.

We analyzed 143 UPEC isolates from six Latin American countries: Paraguay (39.2%), Brazil (32.9%), Peru (11.2%), Colombia (8.4%), Argentina (6.3%), and Mexico (2.1%) (Figure 3). The temporal distribution of the isolates was as follows: 2018 (9.8%), 2019 (6.3%), 2020 (8.4%), 2021 (30.1%), 2022 (44.1%), and 2023 (1.4%). The most frequent UPEC sequence types in Latin America were ST131 (42.7%), ST1193 (13.3%), ST648 (8.4%) and ST998 (3.5%). In Peru, the predominant sequence types were ST131 (43.8%) and ST1193 (37.5%); in Brazil, ST131 (40.4%), ST648 (14.9%) and ST127 (10.6%); in Paraguay, ST131 (39.2%), ST1193 (12.5%) and ST73 (7.5%); in Argentina, ST131 (33.3%) and ST1193 (33.3%); in Colombia, ST131 (66.7%) and ST648 (16.7%); and in Mexico no prominent clones were identified (Figure 3). The most frequent serotype was O25:H4 (43.4%), followed by O75:H5 (11.2%), O1:H6 (6.3%) and O2:H6 (3.5%). The Clermont B2 phylogroup was predominant in this dataset (83.2%), followed by phylogroups F (15.4%) and G (1.4%).

The most common beta-lactamases among the antimicrobial resistance (AMR) genes, were *blaTEM-1* (40.0%), *blaCTX-M-15* (32.2%), *blaCTX-M-27* (9.1%), *blaKPC-2* (4.9%), *blaNDM-1* (2.1%), and *blaKPC* (0.7%). The most common genes associated with aminoglycoside resistance were *aph(6)-Id* (35.0%), *aph(3’’)-Ib* (33.6%), *aadA5* (31.5%), *aac(6’)-Ib-cr5* (22.4*%), and aac(3)-IIe* (16.8%). Among the genes related to efflux pumps, *emrD* was found in all isolates (100%), followed by *acrF* (93.0%) and *mdtM* (60.8%). Other genes also stood out, such as *mphA* (43.4%), associated with macrolide resistance; *sul1* (45.5%) and *sul2* (37.1%), associated with sulfonamide resistance; *tetA* (39.9%) and *tetB* (16.8%), related to tetracycline resistance; and *dfrA17* (36.4%), associated with trimethoprim resistance.

Non-synonymous mutations associated with RAM were found in 69.9% of UPEC isolates, with at least one mutation in the *gyrA* gene, which is related to fluoroquinolone resistance. We found that 67.8% of isolates had double mutations in amino acids Ser-83 and Asp-87 of *gyrA*. Regarding the *parC* gene, also associated with fluoroquinolone resistance, 69.2% had at least one mutation, while 46.9% had double mutations in combinations of the amino acids Ala-108, Ala-56, Glu-84, Ser-57, or Ser-80. Regarding the *pmrB* gene, 79.0% of the isolates had at least one mutation related to colistin resistance, and 1.4% had double mutations between the amino acids Glu-123, Pro-94, Val-161, or Tyr-358. Other genes associated with resistance presented single mutations: 86.0% of isolates had a mutation in the *glpT* gene (Glu-448), 69.2% in *uhpT* (Glu-350), 46.2% in *ptsI* (Val-35), and 16.8% in *cyaA* (Ser-352), all associated with fosfomycin resistance. Besides, 72.0% had mutations in *parE*, which confers resistance to fluoroquinolones, frequently in the amino acids Ile-529 (46.2%) and Leu-416 (13.3%). The mutation in *marR* (Ser-3), associated with multidrug resistance to penicillin, phenols, quinolones, rifamycins, and tetracyclines, was identified in 24.5% of the isolates.

Patterns of coincidence between sequencing type, serotype, and point mutations associated with AMR were identified in the 143 analyzed genomes (Table 1). The most frequent pattern includes ST131 with serotype O25:H4 (35.7%), followed by ST1193 with serotype O75:H5 (11.1%). The three most frequent patterns include the point mutations *gyrA* (D87N), *gyrA* (S83L), and *parC* (S80I), all related to fluoroquinolone resistance. However, *glpT* (E448K), *parC* (E84V), *parE* (I529L), *pmrB* (E123D), *ptsI* (V25I), and *uhpT* (E350Q) were also found in pattern 1; *marR* (S3N), *parE* (L416F), *pmrB* (E123D), *uhpT* (E350Q) in pattern 2; and, *cyaA* (S352T), *glpT* (E448K), *parE* (S458A) in pattern 3. Likewise, patterns 2 and 4 had mutations associated with the highest number of antibiotic classes (seven classes) due to the presence of the MDR *marR* gene. In addition, patterns 1, 2, and 4 were more frequent in isolates from Paraguay, and pattern 3 in isolates from Brazil (Table 1).

**Table 1:** *In silico* prediction of serotype, sequence type, and point mutations associated with resistance in uropathogenic *E. coli* (UPEC) genomes, 2018-2023 (n=143).

Pattern number	Sequence type	Serotype	Mutations associated with antimicrobial resistance	Types of antibiotics	Number of UPEC isolates (%)	Countries (n)
1	131	O25:H4	*glpT*(E448K), *gyrA*(D87N), *gyrA*(S83L), *parC*(E84V), *parC*(S80I), *parE*(I529L), *pmrB*(E123D), *ptsI*(V25I), *uhpT*(E350Q)	3	51 (35.7)	Paraguay (19) Brazil (16) Colombia (8) Peru (6) Argentina (1) Mexico (1)
2	1193	O75:H5	*gyrA*(D87N), *gyrA*(S83L), *marR*(S3N), *parC*(S80I), *parE*(L416F), *pmrB*(E123D), *uhpT*(E350Q)	7	16 (11.1)	Paraguay (7) Peru (6) Argentina (2) Brazil (1)
3	648	O1:H6	*cyaA*(S352T), *glpT*(E448K), *gyrA*(D87N), *gyrA*(S83L), *parC*(S80I), *parE*(S458A)	2	7 (4.9)	Brazil (4) Argentina (1) Colombia (1) Paraguay (1)
4	998	O2:H6	*glpT*(E448K), *marR*(S3N), *pmrB*(E123D)	7	5 (3.5)	Paraguay (3) Argentina (1) Brazil (1)

**Figure 4:**
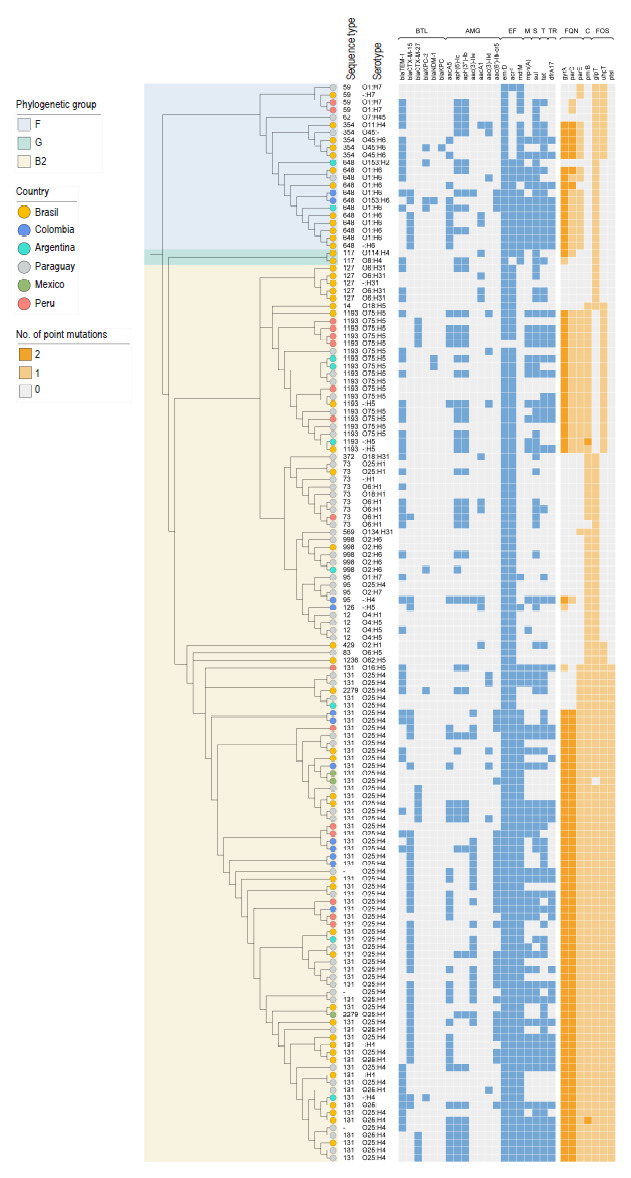
Phylogenetic tree of 143 uropathogenic *E. coli* (UPEC) isolates from Latin America during 2018 and 2023.

Phylogenetic analysis identified two main clades according to the Clermont classification: F and G/B2 (Figure 4). The second clade is larger and is divided into a subclade for group G and two large subclades for B2. Clones ST131, ST1193, ST998, and ST127 are restricted to phylogroup B2, while ST648 and ST354 are restricted to phylogroup F, and ST117 is restricted to phylogroup G. Serotypes O25:H4, O75:H5, and O2:H6 are restricted to phylogroup B2, while O1:H6 and O45:H6 are restricted to phylogroup F. It should be noted that all isolates belonging to ST1193 have a double mutation in the *gyrA* gene and most isolates belonging to ST113 have a double mutation in the *gyrA* and *parC* genes simultaneously.

BTL: beta-lactamases, AMG: aminoglycosides, EF: efflux, M: macrolides, S: sulfonamides, T: tetracycline, TR: trimethoprim, FQN: fluoroquinolones, C: colistin, FOS: fosfomycin

## DISCUSSION

This study analyzed 143 UPEC genomes from six Latin American countries, with a predominance of isolates from Paraguay and Brazil. A high frequency of the ST131 clone (42.7%) was identified, mainly related to serotype O25:H4 and phylogroup B2, followed by ST1193 and ST648. Consistent patterns were found between clone, serotype, and mutation, with frequent combinations of resistance to fluoroquinolones and fosfomycin. Phylogenetic analysis revealed the grouping of the dominant clones within phylogroup B2, while others such as ST648 were restricted to phylogroup F, evidencing the genetic diversity and possible regional adaptation mechanisms of UPEC in Latin America.

During the 2018-2023 period, the ST131 clone was identified in 42.7% of the analyzed UPEC genomes. In comparison, a study conducted in Saudi Arabia in 2020 reported a prevalence of 61.7% ^19^. ST131 is known to be a high-risk, multidrug-resistant (MDR) pandemic strain and one of the leading causes of difficult-to-treat UTIs and bacteremia. This clone has plasmids that encode additional resistance and virulence genes, facilitating its spread in community and hospital settings ^20^. The emerging ST1193 clone, also pandemic and MDR, has also been identified as a cause of UTI and bacteremia ^17^. In our study, 13.3% of isolates from the region correspond to ST1193, and in Peru we identified it in 37.5% of cases. This is higher than the 6% reported in Spain in a collection of UPEC from women in primary care centers ^21^.

Phylogenetic group B2, recognized as the most virulent and highly prevalent in mammals, is associated with persistent extraintestinal infections ^22^. In our study, 83.2% of isolates belonged to group B2, with ST131 clones with serotype O25 standing out. A study in Iraq identified that 33.9% of UPEC belonged to phylogroup B2, of which 92.1% corresponded to ST131 and 97.1% were serotype O25 ^15^. The O25-B2-ST131 lineage is considered hypervirulent, MDR, and ESBL-producing, underscoring the need to implement control measures to limit its spread ^23^. Similarly, a report from Saudi Arabia identified a prevalence of 61.7% for phylogroup B2, with 100% of ST131 clones belonging to this group, a result consistent with our study ^19^. In contrast, a previous study in Peru did not identify phylogroups F and G among UPEC isolates ^24^.

With regard to beta-lactamases, our study highlights the presence of the *blaCTX-M-15* (32.2%) and *blaCTX-M-27* (9.1%) genes. These results differ from a previous study conducted in Peru, which used conventional PCR and reported a frequency of 18% for the *blaCTX* gene, with a different distribution in which *blaCTX-M-1* (72.4%) and *blaCTX-M-9* (25.9%) predominated ^8^. These findings underscore the importance of reporting specific beta-lactamase alleles to better understand their distribution and impact on antimicrobial resistance.

When it comes to genes associated with efflux pumps, we found that 100% of the analyzed genomes contained the *emrD* gene and 93.0% contained the *acrF* gene, both of which play a key role in resistance to multiple families of antibiotics through transcriptional regulation ^13^. These findings are similar to those reported by a study on UPEC in Iraq, which found a prevalence of 100% for *emrD* and 66% for *acrF*^25^. In addition, the *tetA* gene (39.9%) stood out in resistance to tetracyclines, while *sul1* showed a prevalence of 45.5% in resistance to sulfonamides. In contrast, a study in Iran reported that the *tetB* (66.7%) and *sul1* (45.5%) genes were the most prevalent ^26^, which could be attributed to geographical differences and the use of molecular techniques for gene identification.

Single nucleotide polymorphisms (SNPs) also play a crucial role in AMR. In Iran, 91.2% of strains with double mutations in *gyrA* (codons 83 and 106) were found to be resistant to fluoroquinolones, with minimum inhibitory concentration (MIC) values of up to 256 µg/mL ^10^. In our study, 69.9% of isolates had mutations in the *gyrA* gene, and 67.8% had double mutations (codon 83 and 87). In addition, all ST1193 isolates had double mutations in *gyrA*, which is consistent with a study conducted in the United States indicating that this clone is resistant to fluoroquinolones ^27^. In addition, we found that the frequency of double mutations is higher than in other regions, which could be a consequence of the unregulated use of antibiotics in Latin America.

Mutations in *pmrB*, which affect the PmrAB stress response system in enterobacteria, are associated with changes in lipopolysaccharide (LPS), thereby reducing the efficacy of colistin in *mcr*-negative *E. coli* isolates. This effect manifests as a significant increase in MIC to 8 or 16 µg/mL ^12^. Although colistin is not recommended as a treatment for UTIs in the region, an increase in the detection of resistant strains in patients with UTIs has been reported over the last decade ^28^.

One of the main limitations of this study was the small number of analyzed *E. coli* genomes, originating from six Latin American countries, as this may not reflect the actual diversity of UPEC in the region. The years of isolation are not homogeneous across countries, which could influence the reported genomic representativeness. In addition, not all genomes available in the NCBI database are UPEC, despite being classified as UTI by the authors. Finally, although the bioinformatic tools we used are robust and frequently used in genomic studies of AMR, they are constantly updated and may not detect mutations in resistance genes that are clinically relevant in the future.

One of the main strengths of our study lies in the use of a functional definition based on virulence genes to classify UPEC, which provides a solid foundation for future research. This approach not only lays the groundwork for the application of more robust epidemiological designs but, when combined with molecular tools, will enable long-term monitoring with broader and more systematic sampling across different regions and time periods. This will lead to a deeper understanding of the evolution of antimicrobial resistance and the dynamics of UPEC infections, enabling more effective strategies for their control and prevention.

In conclusion, our study identified a high frequency of high-risk uropathogenic *E. coli* clones such as ST131 and ST1193, along with a high frequency of mutations in genes associated with multidrug resistance. These findings underscore the importance of these clones in the epidemiology of UTIs as a public health risk. Therefore, empirical treatment regimens for UTIs should be improved and antimicrobial resistance control policies strengthened. Furthermore, the patterns we report suggest possible clonal spread between countries, highlighting the need for coordinated genomic surveillance efforts at the regional level.

## References

[1] Rodriguez-Angeles M (2002). Principales características y diagnóstico de los grupos patógenos de Escherichia coli. Salud Pública México.

[2] Agarwal J, Srivastava S, Singh M (2012). Pathogenomics of uropathogenic Escherichia coli. Indian J Med Microbiol.

[3] Spurbeck RR, Dinh PC, Walk ST, Stapleton AE, Hooton TM, Nolan LK (2012). Escherichia coli Isolates That Carry vat, fyuA, chuA, and yfcV Efficiently Colonize the Urinary Tract. Infect Immun.

[4] Yang X, Chen H, Zheng Y, Qu S, Wang H, Yi F (2022). Disease burden and long-term trends of urinary tract infections A worldwide report. Front Public Health.

[5] Whelan S, Lucey B, Finn K (2023). Uropathogenic Escherichia coli (UPEC)-Associated Urinary Tract Infections The Molecular Basis for Challenges to Effective Treatment. Microorganisms.

[6] Terlizzi ME, Gribaudo G, Maffei ME (2017). UroPathogenic Escherichia coli (UPEC) Infections Virulence Factors, Bladder Responses, Antibiotic, and Non-antibiotic Antimicrobial Strategies. Front Microbiol.

[7] Kot B (2019). Antibiotic Resistance Among Uropathogenic Escherichia coli. Pol J Microbiol.

[8] Loyola S, Concha-Velasco F, Pino-Dueñas J, Vasquez-Luna N, Juarez P, Llanos C (2021). Antimicrobial Resistance Patterns and Dynamics of Extended-Spectrum ß-Lactamase-Producing Uropathogenic Escherichia coli in Cusco, Peru. Antibiotics.

[9] Pavez M, Troncoso C, Osses I, Salazar R, Illesca V, Reydet P (2019). High prevalence of CTX-M-1 group in ESBL-producing enterobacteriaceae infection in intensive care units in southern Chile. Braz J Infect Dis Off Publ Braz Soc Infect Dis.

[10] Shenagari M, Bakhtiari M, Mojtahedi A, Atrkar Roushan Z (2018). High frequency of mutations in gyrA gene associated with quinolones resistance in uropathogenic Escherichia coli isolates from the north of Iran. Iran J Basic Med Sci.

[11] Garallah ET, Al-Jubori SS (2020). Molecular detection of glpT and uhpT genes as fosfomycin pathways in UTI infection patients. Gene Rep.

[12] Lin JC, Kristopher Siu LK, Chang FY, Wang CH (2024). Mutations in the pmrB gene constitute the major mechanism underlying chromosomally encoded colistin resistance in clinical Escherichia coli. J Glob Antimicrob Resist.

[13] Yu L, Li W, Xue M, Li J, Chen X, Ni J (2020). Regulatory Role of the Two-Component System BasSR in the Expression of the EmrD Multidrug Efflux in Escherichia coli. Microb Drug Resist Larchmt N.

[14] Bunduki GK, Heinz E, Phiri VS, Noah P, Feasey N, Musaya J (2021). Virulence factors and antimicrobial resistance of uropathogenic Escherichia coli (UPEC) isolated from urinary tract infections a systematic review and meta-analysis. BMC Infect Dis.

[15] Al-Guranie DR, Al-Mayahie SM (2020). Prevalence of E coli ST131 among Uropathogenic E. coli Isolates from Iraqi Patients in Wasit Province, Iraq. Int J Microbiol.

[16] Kudinha T, Johnson JR, Andrew SD, Kong F, Anderson P, Gilbert GL (2013). Escherichia coli Sequence Type 131 as a Prominent Cause of Antibiotic Resistance among Urinary Escherichia coli Isolates from Reproductive-Age Women. J Clin Microbiol.

[17] Pitout J, Peirano G, Chen L, DeVinney R, Matsumura Y (2022). Escherichia coli ST1193: Following in the Footsteps of E. coli ST131. Antimicrob Agents Chemother.

[18] Marcos-Carbajal P, Salvatierra G, Yareta J, Pino J, Vásquez N, Diaz P (2021). Caracterización microbiológica y molecular de la resistencia antimicrobiana de Escherichia coli uropatógenas de hospitales públicos peruanos. Rev Peru Med Exp Salud Pública.

[19] Alqasim A, Abu Jaffal A, Alyousef A (2020). Prevalence and molecular characteristics of sequence type 131 clone among clinical uropathogenic Escherichia coli isolates in Riyadh, Saudi Arabia. Saudi J Biol Sci.

[20] Whitmer GR, Moorthy G, Arshad M (2019). The pandemic Escherichia coli sequence type 131 strain is acquired even in the absence of antibiotic exposure. PLoS Pathog.

[21] García-Meniño I, Lumbreras P, Lestón L, Álvarez-Álvarez M, García V, Hammerl JA (2022). Occurrence and Genomic Characterization of Clone ST1193 Clonotype 14-64 in Uncomplicated Urinary Tract Infections Caused by Escherichia coli in Spain. Microbiol Spectr.

[22] Nowrouzian FL, Wold AE, Adlerberth I (2005). Escherichia coli Strains Belonging to Phylogenetic Group B2 Have Superior Capacity to Persist in the Intestinal Microflora of Infants. J Infect Dis.

[23] Shahbazi R, Salmanzadeh-Ahrabi S, Aslani MM, Alebouyeh M, Falahi J, Nikbin VS (2023). The genotypic and phenotypic characteristics contributing to high virulence and antibiotics resistance in Escherichia coli O25-B2-ST131 in comparison to non- O25-B2-ST131. BMC Pediatr.

[24] Rodriguez AOG, Pastor HJB, Villafuerte CAG, Barrón de YLM, Miranda de DVHC, Cunza SS (2019). Clasificación filogenética de Escherichia coli uropatógena y respuesta inmunometabólica en adultos mayores con infección urinaria en casas de reposo. Arch Med Col.

[25] Ali SA, Al-Dahmoshi HOM (2022). Detection of Efflux Pumps Gene and Relation with Antibiotics Resistance in Uropathogenic Escherichia coli (UPEC) Isolated from Patients with Cystitis. Iraqi J Sci.

[26] Boroumand M, Naghmachi M, Ghatee MA (2021). Detection of Phylogenetic Groups and Drug Resistance Genes of Escherichia coli Causing Urinary Tract Infection in Southwest Iran. Jundishapur J Microbiol.

[27] Tchesnokova V, Radey M, Chattopadhyay S, Larson L, Weaver JL, Kisiela D (2019). Pandemic fluoroquinolone resistant Escherichia coli clone ST1193 emerged via simultaneous homologous recombinations in 11 gene loci. Proc Natl Acad Sci.

[28] Zakaria AS, Edward EA, Mohamed NM (2021). Genomic Insights into a Colistin-Resistant Uropathogenic Escherichia coli Strain of O23 H4-ST641 Lineage Harboring mcr-1.1 on a Conjugative IncHI2 Plasmid from Egypt. Microorganisms.

