# Supplementary material for: Genomic diversity of uropathogenic Escherichia coli in clinical isolates from six latin american countries, 2018-2023
Source: Rev Peru Med Exp Salud Publica. 2025 Jun 19;42(2):156–65. doi: 10.17843/rpmesp.2025.422.14299 (PMC12377891; doi:10.17843/rpmesp.2025.422.14299)
Supplement: Translation

# Translation: Diversidad genómica de *Escherichia coli* uropatógena en aislados clínicos de seis países de Latinoamérica, 2018-2023

**Authors:** Francesca Caballero, Anne Martinez-Ventura, Diego Cuicapuza, Alex Fajardo-Loyola, Rosmery Gutierrez-Ajalcriña, Javier Soto-Pastrana, Percy Asmat-Marrufo, Evelyn Barco-Yaipen de Vera, Henry Meza-Fernandez, Mario Chambi-Quispe, Jimena Pino-Dueñas, Nicomedes Laura-Rivas, Alexander Briones-Alejo, Pilar Diaz-Rengifo, Carlos Peralta-Siesquen, Guillermo Salvatierra, Pablo Tsukayama, Pool Marcos-Carbajal

**Affiliations:** 1 Universidad Peruana Cayetano Heredia, Laboratorio de Genómica Microbiana, Lima, Perú.; 2 Emerge (Emerging Diseases and Climate Change Research Unit), Facultad de Salud Pública y Administración, Universidad Peruana Cayetano Heredia, Lima, Perú.; 3 Facultad de Medicina, Universidad Peruana Cayetano Heredia, Lima, Perú.; 4 Instituto Nacional de Salud, Centro Nacional de Salud Pública, Laboratorio de Biotecnología y Biología molecular, Lima, Perú.; 5 Hospital de Huaycán, Área de Epidemiología, Lima, Perú.; 6 Hospital Nacional Docente Madre Niño San Bartolomé, Departamento de Patología Clínica, Área de Microbiología, Lima, Perú.; 7 Laboratorio de Referencia Regional Salud Pública, Servicio de microbiología, Trujillo, La Libertad, Perú.; 8 Hospital JAMO, Área de microbiología, Tumbes, Perú.; 9 Hospital Alberto Sabogal Sologuren, Departamento de Patología Clínica, Servicio de Microbiología, Bellavista, Callao, Perú.; 10 Hospital Carlos Monge Medrano, Patología Clínica, Puno, Perú.; 11 Hospital Regional del Cusco, Patología Clínica, Cusco, Perú.; 12 Laboratorio de Referencia Regional de Salud Pública Huancavelica, Servicio de microbiología, Huancavelica, Perú.; 13 Hospital Regional de Loreto, Servicio de microbiología, Iquitos, Perú.; 14 Laboratorio de Referencia Regional Salud Pública San Martín, Servicio de Microbiología, Tarapoto, Perú.; 15 IPRESS Jorge Chávez, Área de microbiología, Madre de Dios, Perú.; 16 Instituto de Medicina Tropical Alexander von Humboldt, Universidad Peruana Cayetano Heredia, Lima, Perú.; 17 Universidad Peruana Unión, Escuela Profesional Medicina, Laboratorio de Investigación en Biología Molecular, Lima, Perú.

**Keywords:** Escherichia coli, Uropatógeno, UPEC, Resistencia Bacteriana, Epidemiología Molecular

## Abstract

**Objetivo.:**

Caracterizar genéticamente aislados clínicos de *Escherichia coli* uropatógena (UPEC) provenientes de hospitales en Perú y contextualizarlos frente a 127 genomas adicionales de UPEC reportados en seis países de Latinoamérica entre 2018 y 2023.

**Materiales y métodos.:**

Se secuenciaron y ensamblaron los genomas de 16 aislados peruanos de UPEC y se complementaron con 127 genomas disponibles en la base de datos pública del NCBI. Se identificaron serotipos, secuenciotipos (ST), genes de resistencia antimicrobiana (RAM) y mutaciones asociadas a resistencia. Asimismo, se realizó un análisis filogenético para determinar relaciones evolutivas y distribución en filogrupos.

**Resultados.:**

El clon ST131 fue el más prevalente (42,7%), seguido de ST1193 (13,3%). El filogrupo B2 predominó ampliamente (83.2%), destacando el serotipo O25:H4. Se identificaron con alta frecuencia los genes de resistencia *blaTEM-1, blaCTX-M-15* y *blaCTX-M-27,* así como mutaciones en *gyrA* y *parC* asociadas a resistencia a fluoroquinolonas, especialmente en el clon ST131.

**Conclusión.:**

Los hallazgos evidencian una alta circulación de clones de UPEC de alto riesgo, como ST131 y ST1193, en Latinoamérica, junto con una notable carga de genes y mutaciones vinculadas a resistencia a múltiples antimicrobianos, lo que resalta la necesidad de fortalecer la vigilancia genómica regional.

## INTRODUCCIÓN

*Escherichia coli* es un bacilo gramnegativo de la familia *Enterobacteriaceae* que forma parte de la microbiota intestinal normal en la mayoría de los humanos. Sin embargo, existen variantes oportunistas, conocidas como *E. coli* uropatógenas (UPEC, por sus siglas en inglés), capaces de colonizar el tracto urinario y causar infecciones ^1^^,^^2^. Estas cepas UPEC se distinguen por la presencia de al menos tres factores de virulencia específicos: *chuA* y *fyuA*, implicados en la adquisición de hierro; *vat*, que codifica una toxina autotransportadora inductora de vacuolización; y *yfcV*, asociado con fimbrias que facilitan la adhesión a células uroteliales ^3^.

La UPEC es la principal causa de las infecciones del tracto urinario (ITU) a nivel mundial, afectando aproximadamente a 400 millones de personas al año y causando cerca de 230 000 muertes anuales, según el estudio Global Burden of Disease de 2019 ^4^. Estas infecciones constituyen el segundo tipo más común en adultos ^5^, y se clasifican en complicadas (cITU), cuando hay comorbilidades (embarazo, estado inmunocomprometido) o anormalidades del tracto urinario (obstrucción, hidronefrosis, cálculos renales), y no complicadas (uITU), en ausencia de estas condiciones ^6^. Para el tratamiento de las uITU, se emplean antibióticos como ampicilina, sulfametoxazol y ciprofloxacina, mientras que para las cITU se recomiendan nitrofuranos, cefalosporinas y carbapenémicos ^5^.

El uso inadecuado y la escasa regulación en la venta de antibióticos de amplio espectro han contribuido significativamente al aumento de la resistencia antimicrobiana (RAM), especialmente en países de bajos y medianos ingresos ^7^. En UPEC, los mecanismos de RAM más importantes incluyen la producción de betalactamasas de espectro extendido (BLEE), enzimas capaces de hidrolizar penicilinas, betalactámicos y cefalosporinas ^8^. Entre estas enzimas, destaca *blaCTX-M-1*, capaz de hidrolizar cefalosporinas de tercera generación y una de las betalactamasas más frecuentes en *E. coli* productora de BLEE ^9^. Además, existen mecanismos de resistencia no enzimáticos, como las mutaciones puntuales en genes diana: *parC/E* y *gyrA/B* están asociadas a la resistencia a fluoroquinolonas ^10^, *glpT* y *uhpT* a fosfomicina ^11^ y *pmrA/B/D* a colistina ^12^. Otros mecanismos no enzimáticos incluyen las bombas de eflujo, como las codificadas por los genes *emrD*, *acrF* y *mdtM*, que están relacionadas con la multidrogoresistencia (MDR, por sus siglas en inglés) ^13^. Entre el 2000 y 2019, en Europa, Asia y América, se observó una alta resistencia fenotípica a quinolonas (49,4%), betalactámicos (36,9%), aminoglucósidos (28,7%) y fosfomicina (8,4%) ^14^.

Los avances en genómica de las últimas décadas permiten hoy la identificación precisa de secuenciotipos (STs) y clones de alto riesgo. Entre ellos, destaca ST131, el clon MDR de mayor riesgo a nivel mundial ^15^. En Australia, en 2013, se encontró que ST131 (27%) y el serotipo O25b (85%) fueron los más frecuentes en aislados urinarios de una muestra de mujeres en edad reproductiva ^16^. En 2022, en Estados Unidos, se observó que el 23% de los aislados de *E. coli* pertenecían al clon pandémico emergente ST1193, caracterizado por su resistencia a fluoroquinolonas, y su prevalencia fue del 51% en China ^17^.

La falta de información sobre la diversidad genómica y los patrones de resistencia de UPEC en Latinoamérica dificulta su monitoreo y tratamiento. La mayoría de los estudios se limitan a identificar marcadores de linajes y genes de resistencia utilizando PCR convencional. Aunque la secuenciación del genoma completo es una herramienta clave para la caracterización de aislados con alta resolución y a gran escala, hasta la fecha se han reportado pocos estudios en la región que empleen esta técnica para UPEC. Por lo tanto, con el fin de entender mejor la diversidad genómica de este patógeno en Latinoamérica, el objetivo de la presente investigación es caracterizar 16 aislados de UPEC de hospitales en Perú y analizarlos en el contexto de 127 genomas de UPEC reportados en seis países de la región entre 2018 y 2023.

**MENSAJE CLAVE**

Motivación para el estudio. Contribuir a la vigilancia genómica de UPEC en muestras clínicas de Latinoamérica, en respuesta al creciente problema de salud pública representado por las ITU y su resistencia a los antimicrobianos.

Principales hallazgos. El estudio reveló una alta frecuencia de clones de alto riesgo, como ST131 y ST1193. Se identificaron mutaciones críticas en genes asociados con la resistencia a múltiples antibióticos, incluyendo fluoroquinolonas, betalactámicos y fosfomicina.

Implicancias. Los resultados destacan la urgente necesidad de fortalecer el monitoreo de UPEC en Latinoamérica. Rastrear cepas resistentes e implementar medidas que limiten su propagación, es crucial y tiene un impacto significativo en la eficacia de tratamientos disponibles.

## MATERIALES Y MÉTODOS

### Diseño de estudio

Se realizó un estudio descriptivo. La población de estudio corresponde a 143 aislados de UPEC de Latinoamérica. De estos, 127 corresponden a genomas provenientes de la base de datos pública del NCBI y 16 de pacientes ambulatorios con diagnóstico clínico compatible con infección del tracto urinario (ITU) en hospitales del Perú (figura 1).

**Figura 1:** Diagrama de flujo sobre búsqueda de secuencias genómicas de *E. coli* uropatógenas (UPEC) en la base de datos del NCBI Isolates Browser en Latinoamérica y genomas provenientes de aislados de pacientes con ITU en Perú. Se incluyeron 127 genomas de Latinoamérica provenientes de NCBI y se complementaron con 16 recolectados de hospitales del Perú, con un total de 143 genomas de UPEC disponibles para el análisis

NCBI: National Center for Biotechnology Information, LATAM: Latinoamérica, UPEC: *E. coli* uropatógena, ITU: infección del tracto urinario.

### Secuenciación genómica de aislados de UPEC en Perú

Se analizaron cepas de *E. coli* previamente caracterizadas fenotípicamente recolectadas de pacientes con ITU en Perú en 8 hospitales durante 2018-2019 ^18^. El ADN total se extrajo a partir de 5 ml de cultivo líquido en medio TSB, utilizando el kit Thermo GeneJet Genomic DNA Purification Kit (Thermo Scientific) en un volumen final de 100 µl. La cuantificación del ADN se realizó con el fluorómetro Qubit 4 y el kit dsDNA HS (Thermo Scientific). A partir de los 200 aislados originales, se seleccionaron 48 para secuenciación mediante un muestreo estratificado, priorizando la diversidad en perfiles de resistencia antimicrobiana y la representatividad geográfica de los sitios de recolección (figura 1). Las librerías genómicas se prepararon con el kit NexteraXT (Illumina) y se secuenciaron en un instrumento Illumina MiSeq de la UPCH, utilizando kits MiSeq v2 de 500 ciclos. Las secuencias crudas obtenidas del secuenciamiento (formato Fastq) fueron sometidas a un control de calidad utilizando FastQC v0.12.1 (https://github.com/s-andrews/FastQC). Posteriormente, se empleó el programa fastp v0.23.4 (https://github.com/OpenGene/fastp) para eliminar adaptadores y secuencias de baja calidad (Q<30 y longitud <50 bp), generando archivos R1 y R2 pareados. Finalmente, los reads procesados se ensamblaron *de novo* utilizando parámetros por defecto con SPAdes v3.15.2 (https://github.com/ablab/spades). La calidad de los genomas ensamblados se evaluó con la herramienta QUAST v5.2.0 (https://github.com/ablab/quast) (figura 2). Los fastq y fasta se depositaron en el siguiente BioProject: PRJNA1153025.



Figura 2. Flujograma del análisis bioinformático y filogenético. Se ensamblaron los reads provenientes de pacientes aislados de pacientes de hospitales en Perú y se procesaron en conjunto con los genomas provenientes de NCBI mediante herramientas de tipificación y de filogenia

### Genomas públicos de UPEC en Latinoamérica

Se descargaron genomas ensamblados en formato Fasta desde el NCBI Isolates Browser (https://www.ncbi.nlm.nih.gov/pathogens/isolates/), junto con la metadata asociada, que incluía información relevante como el código identificador del aislado, el código de ensamblaje, el año de obtención y el país de origen. Los criterios de selección en la búsqueda incluyeron el grupo taxonómico (taxgroup_name) «*E. coli* and *Shigella»*; el tipo de aislado (epi_type) «clinical»; la fuente del aislado (isolation_source) «urine»; el hospedero (host) «*Homo sapiens*» y la ubicación geográfica (geo_loc_name) limitada a «Paraguay», Argentina, Colombia, Brasil, Chile, Perú, México, Bolivia, Costa Rica, Cuba, Ecuador, El Salvador, Guatemala, Haití, Honduras, Nicaragua, Panamá, República Dominicana, Uruguay y Venezuela, en el periodo comprendido entre 2016 y 2024. En total, 325 genomas públicos cumplieron con estos criterios (figura 1).

### Refinamiento de selección de los genomas de UPEC

Para confirmar que los aislados de *E. coli* correspondían al tipo UPEC, se incluyeron aquellos genomas que poseían tres o más de los factores de virulencia propuestos por Spurbeck *et al*. ^3^*chuA*, *vat*, *fyuA* y *yfcV*, lo que indica un perfil genético consistente con UPEC. Además, se aplicaron filtros de calidad, requiriendo que las secuencias tuvieran una longitud entre 4,5 y 5,5 millones de pares de bases (Mb), y se excluyeron aquellos genomas que presentaban bases ambiguas. Después de aplicar estos filtros, el número final de genomas públicos considerados fue de 127, a los que se sumaron 16 genomas de UPEC generados en este estudio, resultando en un total de 143 genomas analizados (figura 1).

### Clasificación, identificación de genes y análisis filogenético

Para identificar el serotipo de los aislados, se utilizó la herramienta ABRicate v1.0.1 (https://github.com/tseemann/abricate) con la base de datos EcOH. Los secuenciotipos (ST) se determinaron según el esquema *ecoli_achtman_4*, derivado de siete genes marcadores, utilizando el programa MLST v2.23.0 (https://github.com/tseemann/mlst). La identificación de genes de resistencia antimicrobiana y mutaciones se realizó con AMRFinderPlus v3.12.8 (https://github.com/ncbi/amr, base de datos 2024-05-02.2). Los genes de virulencia fueron identificados con VirulenceFinder v2.0.5 (base de datos 2022-12-02) disponible en el servidor del Center for Genomic Epidemiology (CGE) (https://cge.food.dtu.dk/services/VirulenceFinder/). Además, el filogrupo se identificó según el esquema de Clermont utilizando EzClermont v0.7.0 (https://github.com/nickp60/EzClermont). Todos los análisis emplearon un umbral de corte del 90% para cobertura e identidad. Los genomas ensamblados se anotaron utilizando Bakta v1.6.1 (https://github.com/oschwengers/bakta) y se realizó un alineamiento del *core genome* con Panaroo v1.2.10 (https://github.com/gtonkinhill/panaroo). Posteriormente, se determinó el mejor modelo para la elaboración del árbol filogenético mediante ModelTest-NG v0.1.7 (https://github.com/ddarriba/modeltest). Finalmente, se construyó el árbol utilizando el modelo GTR+I+G4 y realizando 100 réplicas de bootstrap con RAxML-NG v1.2.0 (https://github.com/amkozlov/raxml-ng). Se usó un genoma de *Escherichia fergusonii* (CP083638.1) para enraizar el árbol. La anotación y visualización del árbol se realizaron con iTOL v6 (https://itol.embl.de/) (figura 2).

### Consideraciones éticas

El estudio fue evaluado y aprobado por el Comité de Ética de la Universidad Peruana Unión (N2019-CEUPeU-0001) y por la Comité Institucional de Ética en Investigación (CIEI) de la Universidad Peruana Cayetano Heredia (SIDISI N°214524 y N°214927). Se trabajó únicamente con aislados bacterianos, sin incluir ni analizar información personal o clínica de los pacientes.

## RESULTADOS

Se analizaron 143 aislados de UPEC de seis países de Latinoamérica: Paraguay (39,2%), Brasil (32,9%), Perú (11,2%), Colombia (8,4%), Argentina (6,3%) y México (2,1%) (figura 3). La distribución temporal de los aislados fue la siguiente: 2018 (9,8%), 2019 (6,3%), 2020 (8,4%), 2021 (30,1%), 2022 (44,1%) y 2023 (1,4%). Los secuenciotipos más frecuentes de UPEC en Latinoamérica fueron ST131 (42,7%), ST1193 (13,3%), ST648 (8,4%) y ST998 (3,5%). En Perú, los secuenciotipos predominantes fueron ST131 (43.8%) y ST1193 (37,5%); en Brasil, ST131 (40,4%), ST648 (14,9%) y ST127 (10,6%); en Paraguay, ST131 (39,2%), ST1193 (12,5%) y ST73 (7,5%); en Argentina, ST131 (33,3%) y ST1193 (33,3%); en Colombia, ST131 (66,7%) y ST648 (16,7%); y en México no se identificaron clones destacados (figura 3). El serotipo más frecuente fue O25:H4 (43,4%), seguido por O75:H5 (11,2%), O1:H6 (6,3%) y O2:H6 (3,5%). El filogrupo Clermont B2 fue predominante en este set de datos (83,2%), seguido por los filogrupos F (15,4%) y G (1,4%).

**Figura 3:** Distribución geográfica de clones de *E. coli* uropatógenas (UPEC) en Latinoamérica.

Entre los genes de resistencia antimicrobiana (RAM), las betalactamasas más frecuentes fueron *blaTEM-1* (40,0%), *blaCTX-M-15* (32,2%), *blaCTX-M-27* (9,1%), *blaKPC-2* (4,9%), *blaNDM-1* (2,1%) y *blaKPC* (0,7%). Los genes asociados a la resistencia a aminoglucósidos más comunes fueron *aph(6)-Id* (35,0%), *aph(3’’)-Ib* (33,6%), *aadA5* (31,5%), *aac(6’)-Ib-cr5* (22,4%) y *aac(3)-IIe* (16,8%). Entre los genes relacionados con bombas de eflujo, *emrD* estuvo presente en todos los aislados (100%), seguido por *acrF* (93,0%) y *mdtM* (60,8%). Además, destacaron otros genes como *mphA* (43,4%), asociado con resistencia a macrólidos; *sul1* (45,5%) y *sul2* (37,1%), asociados a la resistencia a sulfonamidas; *tetA* (39,9%) y *tetB* (16,8%), relacionados con la resistencia a tetraciclina; y *dfrA17* (36,4%), asociado a la resistencia a trimetoprima.

Se observaron mutaciones no sinónimas asociadas a RAM en el 69,9% de los aislados de UPEC, presentando al menos una mutación en el gen *gyrA*, relacionado con la resistencia a fluoroquinolonas. El 67,8% de los aislados presentaron mutaciones dobles en los aminoácidos Ser-83 y Asp-87 de *gyrA*. En el gen *parC*, también asociado a la resistencia a fluoroquinolonas, el 69.2% presentó al menos una mutación, mientras que el 46,9% presentó mutaciones dobles en combinaciones entre los aminoácidos Ala-108, Ala-56, Glu-84, Ser-57 o Ser-80. Respecto al gen *pmrB*, el 79,0% de los aislados tuvo al menos una mutación relacionada con la resistencia a colistina, y el 1,4% presentó mutaciones dobles entre los aminoácidos Glu-123, Pro-94, Val-161 o Tyr-358. Otros genes asociados a resistencia presentaron mutaciones únicas: el 86.0% de los aislados presentó mutación en el gen *glpT* (Glu-448), el 69,2% en *uhpT* (Glu-350), el 46,2% en *ptsI* (Val-35) y el 16.8% en *cyaA* (Ser-352), todas asociadas a la resistencia a fosfomicina. El 72,0% presentó mutaciones en *parE*, que confiere resistencia a fluoroquinolonas, frecuentemente en los aminoácidos Ile-529 (46,2%) y Leu-416 (13,3%). La mutación en *marR* (Ser-3), asociada con multidrogoresistencia a penicilinas, fenicoles, quinolonas, rifamicinas, tetraciclinas, se identificó en el 24,5% de los aislados.

Se identificaron patrones de coincidencia entre secuenciotipo, serotipo y mutaciones puntuales asociadas a RAM en los 143 genomas analizados (tabla 1). El patrón más frecuente incluye al ST131 con el serotipo O25:H4 (35,7%), seguido por ST1193 con el serotipo O75:H5 (11,1%). En los tres patrones más frecuentes se encuentran las mutaciones puntuales *gyrA* (D87N), *gyrA* (S83L), *parC* (S80I), todas relacionadas con la resistencia a fluoroquinolonas. Sin embargo, en el patrón 1 también se encontraron *glpT* (E448K), *parC* (E84V), *parE* (I529L), *pmrB* (E123D), *ptsI* (V25I), *uhpT* (E350Q); en el patrón 2, *marR* (S3N), *parE* (L416F), *pmrB* (E123D), *uhpT* (E350Q); y en el patrón 3, *cyaA* (S352T), *glpT* (E448K), *parE* (S458A). Asimismo, los patrones 2 y 4 presentaron mutaciones asociadas con el mayor número de clases de antibióticos (siete clases) debido a la presencia del gen MDR *marR*. Además, los patrones 1, 2 y 4 fueron más frecuentes en los aislados provenientes de Paraguay, y el patrón 3 en los aislados de Brasil (tabla 1).

**Tabla 1:** Predicción in silico de serotipo, secuenciotipo y mutaciones puntuales asociadas a resistencia en genomas de *E. coli* uropatógenas (UPEC), 2018-2023 (n=143).

Número de patrón	Secuenciotipo	Serotipo	Mutaciones asociadas a resistencia antimicrobiana	Clases de antibióticos	Número de aislados de UPEC (%)	Países (n)
1	131	O25:H4	*glpT*(E448K), *gyrA*(D87N), *gyrA*(S83L), *parC*(E84V), *parC*(S80I), *parE*(I529L), *pmrB*(E123D), *ptsI*(V25I), *uhpT*(E350Q)	3	51 (35,7)	Paraguay (19) Brasil (16) Colombia (8) Perú (6) Argentina (1) México (1)
2	1193	O75:H5	*gyrA*(D87N), *gyrA*(S83L), marR(S3N), *parC*(S80I), *parE*(L416F), *pmrB*(E123D), *uhpT*(E350Q)	7	16 (11,1)	Paraguay (7) Perú (6) Argentina (2) Brasil (1)
3	648	O1:H6	*cyaA*(S352T), *glpT*(E448K), *gyrA*(D87N), *gyrA*(S83L), *parC*(S80I), *parE*(S458A)	2	7 (4,9)	Brasil (4) Argentina (1) Colombia (1) Paraguay (1)
4	998	O2:H6	*glpT*(E448K), *marR*(S3N), *pmrB*(E123D)	7	5 (3,5)	Paraguay (3) Argentina (1) Brasil (1)

En el análisis filogenético se identificaron dos clados principales según la clasificación Clermont: F y G/B2 (figura 4). El segundo clado es más extenso y está dividido en un subclado para el grupo G y dos grandes subclados para B2. Se observa que los clones ST131, ST1193, ST998 y ST127 se encuentran restringidos al filogrupo B2, mientras que ST648 y ST354 al filogrupo F, y ST117 al filogrupo G. Los serotipos O25:H4, O75:H5 y O2:H6 están restringidos al filogrupo B2, mientras que O1:H6 y O45:H6 al filogrupo F. Cabe destacar que todos los aislados pertenecientes al ST1193 presentan doble mutación en el gen *gyrA* y la mayoría de aislados pertenecientes al ST113 presentan doble mutación en los genes *gyrA* y *parC* simultáneamente.

**Figura 4:** Árbol filogenético de 143 aislados de *E. coli* uropatógenas (UPEC) de Latinoamérica durante 2018 y 2023.

BTL: betalactamasas, AMG: aminoglucósidos, EF: eflujo, M: macrólidos, S: sulfonamidas, T: tetraciclina, TR: trimeptropim, FQN: fluoroquinolonas, C: colistina, FOS: fosfomicina

## DISCUSIÓN

En este estudio se analizaron 143 genomas de UPEC provenientes de seis países de Latinoamérica, con predominio de aislados de Paraguay y Brasil. Se identificó una alta frecuencia del clon ST131 (42,7%), principalmente relacionado al serotipo O25:H4 y al filogrupo B2, seguido por ST1193 y ST648. Se observaron patrones entre clon, serotipo y mutación consistentes, con presencia frecuente de combinaciones de resistencia a fluoroquinolonas y fosfomicina. El análisis filogenético reveló la agrupación de los clones dominantes dentro del filogrupo B2, mientras que otros como ST648 se restringieron al filogrupo F, lo que evidencia la diversidad genética y los posibles mecanismos de adaptación regional de UPEC en Latinoamérica.

Durante el período 2018-2023, el clon ST131 se identificó en un 42,7% de los genomas UPEC analizados. En comparación, un estudio realizado en Arabia Saudita en 2020 reportó una prevalencia del 61,7% ^19^. ST131 es conocido por ser una cepa pandémica de alto riesgo y multidrogoresistente (MDR), siendo una de las principales causas de ITU y bacteriemias difíciles de tratar. Este clon presenta plásmidos que codifican genes de resistencia y virulencia adicionales, facilitando su diseminación en entornos comunitarios y hospitalarios ^20^. El clon emergente ST1193, también pandémico y MDR, también ha sido identificado como causa de ITU y bacteriemias ^17^. En nuestro estudio, el 13,3% de los aislados de la región corresponden a ST1193, y en Perú se identificó en el 37,5% de los casos. Ello es mayor al 6% reportado en España en una recolección de UPEC en mujeres de centros de atención primaria ^21^.

El grupo filogenético B2, reconocido como el más virulento y con alta prevalencia en mamíferos, está asociado con infecciones extraintestinales persistentes ^22^. En nuestro estudio, el 83,2% de los aislados pertenecen al grupo B2, destacándose los clones ST131 con serotipo O25. Un estudio en Irak identificó que el 33,9% de UPEC pertenecían al filogrupo B2, de los cuales el 92,1% corresponden al ST131 y el 97,1% eran del serotipo O25 ^15^. El linaje O25-B2-ST131 es considerado hipervirulento, MDR y productor de BLEE, lo que subraya la necesidad de implementar medidas de control para limitar su propagación ^23^. De manera similar, un reporte en Arabia Saudita identificó una prevalencia del 61,7% para el filogrupo B2, y que el 100% de los clones ST131 pertenecían a este grupo, resultado consistente con nuestro estudio ^19^. En contraste, un estudio previo en Perú no identificó los filogrupos F y G entre los aislados de UPEC ^24^.

En cuanto a las betalactamasas, nuestro estudio destaca la presencia de los genes *blaCTX-M-15* (32,2%) y *blaCTX-M-27* (9,1%). Estos resultados difieren de un estudio previo realizado en Perú, que utilizó PCR convencional y reportó una frecuencia del 18% para el gen *blaCTX*, con una distribución diferente donde predominaron *blaCTX-M-1* (72,4%) y *blaCTX-M-9* (25,9%) ^8^. Estos hallazgos subrayan la importancia de reportar alelos específicos de betalactamasas para comprender mejor su distribución e impacto en la resistencia antimicrobiana.

En relación con los genes asociados con bombas de eflujo, observamos que el 100% de los genomas analizados contenían el gen *emrD* y el 93,0% el gen *acrF*, los cuales desempeñan un rol fundamental en la resistencia a múltiples familias de antibióticos mediante la regulación transcripcional ^13^. Estos hallazgos son similares a los reportados en un estudio de UPEC en Irak, donde se encontró una prevalencia del 100% para *emrD* y del 66% para *acrF*^25^. Además, el gen *tetA* (39,9%) destacó en la resistencia a tetraciclinas, mientras que *sul1* mostró una prevalencia del 45,5% en la resistencia a sulfonamidas. En contraste, un estudio en Irán reportó que los genes *tetB* (66,7%) y *sul1* (45,5%) fueron los más prevalentes ^26^, lo que podría atribuirse a diferencias geográficas y al uso de técnicas moleculares para la identificación de los genes.

Las mutaciones puntuales (SNPs) también desempeñan un papel crucial en la RAM. En Irán, se encontró que el 91,2% de cepas con dobles mutaciones en *gyrA* (codón 83 y 106) fueron resistentes a fluoroquinolonas, con cifras de concentración inhibitoria mínima (CIM) de hasta 256 µg/mL ^10^. En nuestro estudio, el 69,9% de los aislados presentaron mutaciones en el gen *gyrA*, y el 67,8% presentaron mutaciones dobles (codón 83 y 87). Además, todos los aislados ST1193 tenían mutaciones dobles en *gyrA*, lo que coincide con un estudio realizado en Estados Unidos que indica que este clon es resistente a fluoroquinolonas ^27^. Se puede observar que en nuestro estudio la frecuencia de mutaciones dobles es superior al resto de las regiones, lo que podría ser consecuencia del uso no regulado de antibióticos en Latinoamérica.

Las mutaciones en *pmrB*, que afectan al sistema PmrAB de respuesta al estrés en enterobacterias, se asocian con cambios en el lipopolisacárido (LPS), reduciendo así la eficacia de la colistina en aislados de *E. coli* negativos para *mcr*. Este efecto se manifiesta como una elevación significativa de la CIM a 8 o 16 µg/mL ^12^. Aunque la colistina no se recomienda como tratamiento para ITU en la región, se ha observado un aumento en la detección de cepas resistentes en pacientes con ITU en la última década ^28^.

Entre las limitaciones de este estudio, se debe considerar el reducido número de genomas de *E. coli* analizados, provenientes de seis países de Latinoamérica, lo cual podría no reflejar la diversidad real de UPEC en la región. Los años de aislamiento no son homogéneos entre países, lo que podría influir en la representatividad genómica observada. Además, no todos los genomas disponibles en la base de datos del NCBI son de UPEC, a pesar de estar clasificados como ITU por los autores. Por último, aunque las herramientas bioinformáticas empleadas son robustas y frecuentemente utilizadas en estudios genómicos de RAM, estas se actualizan constantemente y podrían no detectar mutaciones en genes de resistencia que tengan relevancia clínica en el futuro.

Una de las principales fortalezas de este estudio radica en la utilización de una definición funcional basada en genes de virulencia para clasificar UPEC, lo que proporciona una base sólida para futuras investigaciones. Este enfoque no solo sienta las bases para la aplicación de diseños epidemiológicos más robustos, sino que, combinado con herramientas moleculares, permitirá un seguimiento a largo plazo, con un muestreo más amplio y sistemático en diversas regiones y períodos. De esta manera, se logrará una comprensión más profunda de la evolución de la resistencia antimicrobiana y la dinámica de las infecciones por UPEC, permitiendo estrategias más efectivas para su control y prevención.

En conclusión, este estudio identificó una alta frecuencia de clones de *E. coli* uropatógena de alto riesgo como ST131 y ST1193, junto con una elevada frecuencia de mutaciones en genes asociados con la resistencia a múltiples antimicrobianos. Estos hallazgos subrayan la importancia de estos clones en la epidemiología de las ITU como un riesgo para la salud pública. Por lo cual, se deben mejorar los esquemas empíricos de tratamiento de ITU y fortalecer las políticas de control de resistencia antimicrobiana. Además, los patrones encontrados sugieren una posible diseminación clonal entre países, lo que resalta la necesidad de esfuerzos coordinados de vigilancia genómica a nivel regional.
